# Emotional Distress and Body Dissatisfaction: The Mediating Role of Social Media and Emotional Regulation

**DOI:** 10.3390/bs14070580

**Published:** 2024-07-09

**Authors:** Milena López-Montón, Gema Aonso-Diego, Ana Estévez

**Affiliations:** Department of Psychology, Faculty of Health Sciences, University of Deusto, 48007 Bilbo, Spain; milena.lopez@deusto.es (M.L.-M.); gema.aonso@deusto.es (G.A.-D.)

**Keywords:** body dissatisfaction, anxiety, depression, emotion regulation, problematic use of social media

## Abstract

Background: Body dissatisfaction is defined as a negative attitude towards one’s body, characterized by emotional disorders. Currently, problematic use of social media seems to be associated with an increase in body dissatisfaction and emotional distress. Therefore, the present study examined the relationship between emotional distress (i.e., depression, anxiety, and stress) and body dissatisfaction, as well as the mediating role of emotional regulation and problematic social media use in this relationship. In addition, the study aims to identify sex differences in the four research variables. Methods: Measures of the four variables mentioned above were administered to 2520 participants over 18 (*M* = 48.35; *SD* = 16.56, 51% females). Results: The results reveal that women reported higher levels of emotional regulation, emotional distress, body dissatisfaction, and problematic use of social media. Emotional distress correlated with problematic use of social media, emotion regulation strategies (i.e., rumination and catastrophizing), and body dissatisfaction. The relationship between emotional distress and body dissatisfaction was mediated by the problematic use of social media and emotional regulation. Conclusions: These findings underscore the relevance of educating social media users on strategies for emotional regulation. The results highlight the clinical importance of including the emotion regulation approach to a comprehensive understanding of body dissatisfaction.

## 1. Introduction

Body dissatisfaction is an experience that encompasses an individual’s negative thoughts and feelings about their body and appearance. It includes the subjective evaluation of one’s image and the perceived discrepancy between one’s actual and desired physical appearance [1,2]. Body dissatisfaction is not a stable individual trait, but an attitude towards one’s appearance with a cognitive and affective basis and, therefore, it may vary under the influence of different psychological or sociocultural factors [3]. This phenomenon is observed in both women and men [4], may occur across the lifespan, and is exacerbated by social comparison [1,5]. According to the Social Comparison Theory (SCT), first proposed by Leon Festinger in 1954, people evaluate their own opinions, skills, and abilities by comparing them to those of others. It also suggests that such comparison occurs particularly in situations of uncertainty, where it may be difficult to measure one’s abilities objectively [6]. In terms of body image, a person’s level of attractiveness or beauty is currently measured through social comparison, creating a gradation of what is considered a normative image [1]. From the perspective of this theory, people are driven to compare their image and body to others. However, it has been observed that women present more body image problems than men. Women’s most common body image problems are related to the thinness ideal, obsession with weight, and current beauty standards (e.g., small nose, foxy eyes, big lips, and cheekbones) [7]. Indeed, a qualitative study by Kenny et al. found that women tend to imitate the behaviors (e.g., clothing, diet, and skin care) of those they find attractive in their search for acceptance and belonging [8]. In other words, women tend to seek social validation through their physical appearance, which is one of the reasons why women have more problems with body dissatisfaction than men [9,10].

Body dissatisfaction is known to affect physical and mental health. Previous studies have found an association with lower quality of life [11,12], as well as increased depression [13,14], anxiety [15,16], stress [17,18], and body dysmorphia [19]. In addition, greater body dissatisfaction is associated with appearance modification behaviors such as plastic surgery [20,21], cosmetics and unregulated supplementation [22], excessive exercise, and laxative or steroid use [23]. These issues may also play an important etiological role in developing eating disorders (EDs) [24]. Several systematic reviews have highlighted sex differences in the occurrence of EDs. Specifically, 8.4% of women have an ED compared to 2.2% of men [25]. Similarly, 30.3% of women have a dysfunctional pattern of food intake compared to 17% of men [26]. On the other hand, body dissatisfaction is also associated with detrimental health behaviors such as a reduced propensity to self-examine for cancer prevention [27], increased rates of smoking [28], muscle dysmorphia [29], and sexual dysfunction [30].

One of the variables that has recently received the most attention regarding body satisfaction is the use of social media [31]. Despite its benefits, social media has a high potential for addiction and, therefore, may be associated with negative consequences [32,33] such as cyberbullying [34] and pornography use [35]. Nowadays, the problematic use of social media is not included in any of the international classifications of mental disorders, such as the *International Classification of Diseases* (ICD-11) [36] or the *Diagnostic and Statistical Manual of Mental Disorders* (DSM-5-TR) [37]. For this reason, there is no consensus among the scientific community on which terminology to utilize: social media addiction, problematic use of social media, social media abuse, or social media misuse.

Previous studies have shown that people are influenced at the behavioral, emotional, and identity levels by the content they consume on social media [32,38,39]. This influence of social media on behavior and thinking can be harmful, as social media constantly perpetuates and generates unachievable beauty standards (e.g., miracle diets, unsupervised exercise, and untested supplements) [22,40,41], which could affect body satisfaction [1,9,40]. In an online experimental study, Castellanos Silva and Steins exposed 226 individuals to Instagram images related to hegemonic beauty ideals in the experimental group and to body diversity in the control group. The results showed significant group differences, including an increase in body dissatisfaction in the experimental group and a decrease in the control group after exposure [1]. Consistent with these findings, Pedalino and Camerini concluded that Instagram use was associated with lower body satisfaction among young female users, mediated by social comparison with social media influencers. They also found that being a female adolescent and having a higher Body Mass Index (BMI) were associated with lower body satisfaction [9]. This phenomenon has been studied particularly among adolescents and young women, reporting negative effects on body image due to exposure to content depicting thinness ideals and fitness [9,42]. Although most studies have been performed with young women, a recent study found that middle-aged and older women who compare themselves on social media also have image-related problems [10].

In parallel, in the case of men, shirtless images within the muscular ideal have been shown to have an especially negative effect on body satisfaction [43]. As discussed, body image occurs in conjunction with exposure to numerous sociocultural factors (e.g., media, globalization, digitization, physical comparisons, comments, bullying, and social exclusion). All these factors tend to perpetuate body dissatisfaction, a consistent predictor of eating disorders and other mental disorders [24].

One of the variables studied as a protective factor against body dissatisfaction is emotional regulation [44,45]. The construct of emotional regulation refers to the processes by which emotional experiences are evaluated, monitored, maintained, and modified [44,46]. It has also been found that women and men differ in emotional regulation [47]. With respect to the present study, it has been shown that emotional regulation could act as a mediator between emotional well-being and eating behavior [16,41]. Similarly, the literature has shown that emotional regulation is related to problematic social media use [48]. This statement makes sense as difficulties in emotional regulation are related to several psychological disorders, such as anxiety, depression, conduct disorders, and addictive behaviors [42,49], meaning that both problematic social media use and body image problems could be the product of poor emotional regulation. Thus, social media and risky eating behaviors might be strategies for coping with complex emotions such as frustration, inadequacy, and isolation [50,51].

This conclusion leads to the aim of this research: to examine the mediating role of emotional regulation and social media use in the relationship between emotional distress and body dissatisfaction. Based on the assumption that the four variables are linked, it would make sense to delve into the interrelationship of the variables: emotion regulation, social media use, emotional distress, and body dissatisfaction. As noted above, body dissatisfaction is associated with emotional distress, particularly symptoms of anxiety and depression [1,9,13]. Both variables selected as mediators (i.e., emotional regulation and problematic social media use) have been shown to influence mental health and body image. On the one hand, emotional regulation is a protective factor against stressful events. Thus, it improves mental well-being [1,41]. The ability to regulate emotions may protect against beauty standards, stereotypes, and social pressure [16,41]. Hughes and Gullone found that adolescents who reported frequent body image concerns were more likely to have higher levels of depressive symptoms if they used dysfunctional emotion regulation strategies (e.g., avoidance) [44]. On the other hand, problematic social media use has been shown to be a risk factor for mental health. In fact, many authors report negative consequences, especially anxiety, depression, and problems related to body image [1,14,15,16]. For example, Thai et al. showed that reducing social media use improved body image and emotional well-being [39]. It is, therefore, appropriate to examine the mediating role of these two factors: emotional regulation and problematic social media use.

It is true that there are a considerable number of studies that delve into emotional well-being, social media use, emotional regulation, and body dissatisfaction. However, few studies have comprehensively assessed the relationship of the four phenomena holistically. Indeed, when investigating social media and body dissatisfaction, it is not the phenomenon of social media use per se that is studied, but the psychological impact of exposure to images [42,43,52]. Furthermore, most of the studies that have evaluated this phenomenon are carried out with adolescents and young adults (see, e.g., Osorio Cámara et al. [53]; Valencia Ortiz et al. [54]), with a significant scarcity of studies in the adult population. Certainly, in Spain, the younger population is the largest consumer of social media (94.5%), but currently there are 45.1% of users aged 55–64 [55]. Therefore, it would be relevant to avoid ignoring this part of the social media user population and to learn more about the risks and advantages of social media for the adult population. In addition, there are no studies carried out in Spain that investigate the use of social media, emotional regulation, emotional distress, and body dissatisfaction.

Within this background, the aim of this study was threefold: (1) to examine differences between men and women regarding emotional distress, emotional regulation, social media use, and body dissatisfaction; (2) to examine correlations between emotional distress (i.e., depression, anxiety, and stress), emotional regulation (i.e., putting things into perspective, positive refocusing, positive reappraisal, acceptance, refocusing on planning, self-blame, blaming others, rumination, and catastrophizing), problematic social media use (i.e., obsession, lack of control, and excessive use), and body dissatisfaction; and (3) to examine the relationship between emotional distress and body dissatisfaction and the mediating role of emotional regulation and problematic social media use in this relationship.

Based on the above, the hypotheses of this study are the following: (1) concerning sex differences, women will obtain higher scores in emotional distress and the use of social media; (2) all variables, including their dimensions, will be related to each other; (3) the use of social media and emotional regulation are factors involved in body satisfaction and, therefore, in emotional distress.

## 2. Material and Methods

### 2.1. Participants

The study sample comprises 2520 adults (*M* = 48.35, *SD* = 16.56). Forty-nine percent were male (*n* = 1231) and 51% were female (*n* = 1289). The age of the sample was divided into 6 age groups: 18–24 years (*n* = 261), 25–34 years (*n* = 348), 35–44 years (*n* = 489), 45–54 years (*n* = 465), 55–64 years (*n* = 381), and over 65 years (*n* = 576). The sample came from all the representative autonomous communities of Spain. According to employment status, participants can be differentiated into the following groups: currently working (*n* = 1356), retired, pensioner, or disabled (*n* = 655), unemployed (*n* = 234), student (*n* = 188), and housework (*n* = 87). All the participants in the sample were users of at least one of the following social media platforms: Instagram, Facebook, Twitter, TikTok, OnlyFans, Snapchat, and YouTube.

### 2.2. Procedure

For this study, the inclusion criteria were the following: (a) being a resident of Spain, (b) over 18 years of age, (c) completing the entire questionnaire, and (d) correctly answering attention control questions (e.g., “What year is this?”). On the other hand, participants who took less than 30% of the estimated average time to complete the questionnaire (i.e., less than 25–30 min) were excluded.

We informed the participants of the purpose of the study and its approximate duration, and then asked for their consent, assuring them that their data would be kept confidential and that they could withdraw if they wished.

‘Recaptcha’, a system used by Google to detect traffic from automated programs or bots, was used to verify that respondents were real persons. In addition, a control called ‘Relevant ID’ was used to identify users and prevent the same person from taking the survey twice.

All participants completed the informed consent form before completing the survey. This study had the ethical approval of the University of Deusto (ref.: ETK-25/21-22).

### 2.3. Instruments

We used the following measuring instruments:

Initially, questions were asked about socio-demographic data, specifically age, sex, and place of birth.

The *Depression, Anxiety and Stress Scale* (DASS-21; Lovibond & Lovibond [56], adapted into Spanish by Bados et al. [57]) is a 21-item instrument that assesses and discriminates symptoms of anxiety, depression, and stress. It is rated on a 4-point Likert ranging from 0 (*has not happened to me*) to 3 (*has happened to me a lot, or most of the time*). It has three subscales: (1) Stress (α = 0.894); (2) Depressive symptoms (α = 0.921); and (3) Anxious symptoms (α = 0.870). In this study, Cronbach’s alpha for the full scale was 0.955.

The *Social Media Addiction Questionnaire* (ARS, the Spanish acronym for “Adicción a Redes Sociales”; Escurra & Salas [58]) measures social media use and consists of 24 Likert-type scale items that are rated from 1 (*always*) to 5 (*never*). It distinguishes three factors: Obsession with social media (α = 0.910), Lack of personal control in the use of social media (α = 0.504), and Excessive use of social media (α = 0.870). In this study, Cronbach’s alpha for the full scale was 0.96. Higher ARS scores entail greater problematic social media use.

The *Cognitive Emotion-Regulation Questionnaire* (CERQ; Garnefski et al. [59], validated in Spanish by Medrano et al. [60]) was also used. This questionnaire assesses people’s cognitive processes after experiencing a stressful or negative event. For this study, we used an 18-item reduced version [60], rated on a Likert-type scale ranging from 1 (*almost never)* to 5 (*almost always*). The questionnaire has 9 subscales: Rumination (α = 0.751), Catastrophizing (α = 0.903), Self-blaming (α = 0.871), Blaming others (α = 0.873), Putting into perspective (α = 0.751), Acceptance (α = 0.886), Positive Focus (α = 0.829), Positive Reappraisal (α = 0.820), and Refocusing on plans (α = 0.849). For this study, the Cronbach’s alpha of the full questionnaire was 0.860.

Finally, the *Eating Disorders Inventory* (EDI-II; Garner [61], adapted into Spanish by Corral et al. [62]), was used. Only the 9 items of the Body Dissatisfaction subscale, which assesses dissatisfaction with the overall shape and size of body parts of concern in eating disorders (e.g., “I think my stomach is too big”), were used in this study. A six-point Likert-type scale ranging from 1 (*never*) to 6 (*always*) was used. Cronbach’s alpha for the original validation was 0.90, and it was 0.87 for this study.

### 2.4. Data Analysis

The initial dataset contained 2520 pieces of data. First, reliability tests were conducted on the questionnaires (see Instruments section). The Cronbach’s alphas of these questionnaires were all above 0.8, reaching the recommended value of 0.6. (see Instruments section). To test the hypotheses, three data analyses were performed: descriptive analysis, Student’s *t*-tests, Pearson’s r correlations, and mediation analysis. All analyses were carried out using the SPSS package (version 24.0) and confidence levels were set at 95%.

Firstly, descriptive statistics (i.e., mean and standard deviation) were calculated to assess participants’ variables. Secondly, a *t*-test was used to test statistical sex differences in the psychological variables, which are emotional distress (i.e., depression, anxiety, and stress), emotion regulation (i.e., putting into perspective, positive refocusing, positive reappraisal, acceptance, refocusing on planning, self-blame, blaming others, rumination, and catastrophizing), body dissatisfaction, and problematic social media use (i.e., excessive use, obsession, and lack of control). The effect size was calculated using Cohen’s d [63].

Thirdly, this analysis was complemented with Pearson’s r correlations to examine the relationship between the target variables (i.e., emotional distress, emotion regulation, body dissatisfaction, and problematic social media use). For this analysis, both global and subscale scores were considered.

Finally, a mediation analysis was performed using Process model 4 for SPSS Statistics 24, with 10,000 bootstrap samples and 95% bias-corrected intervals to test the mediating role of emotional regulation and problematic social media use in the relationship between emotional distress and body dissatisfaction [64]. Therefore, emotional regulation strategies and problematic social media use were mediation variables in the relationship between emotional distress and body dissatisfaction. Given the high correlations between the emotional distress dimensions (i.e., depression, anxiety, and stress), the problematic social media use dimensions (i.e., excessive use, obsession, and lack of control), and between emotional regulation strategies (i.e., putting into perspective, positive refocusing, positive reappraisal, acceptance, refocusing on planning, self-blame, blaming others, rumination, and catastrophizing), we used the total score of these questionnaires.

## 3. Results

Women showed high rates in all the dimensions of emotional distress. Regarding emotional regulation strategies, whereas most strategies did not show statistically significant sex differences, women displayed a greater tendency towards strategies such as rumination, catastrophizing, and acceptance. Similarly, women reported higher levels of body dissatisfaction. In contrast, concerning problematic use of social media, men obtained higher scores in both the overall score and the subscales of Excessive use, Obsession, and Lack of control (see Table 1).

The correlational results indicated a strong relationship between emotional distress (and its dimensions) and emotion regulation strategies, with special emphasis on the association between anxiety and rumination (*r* = 0.351) and depression and catastrophizing (*r* = 0.470). Furthermore, emotional distress was related to greater problematic use of social media (*r* = 0.416) and greater body dissatisfaction (*r* = 0.347). On the other hand, emotion regulation strategies were directly related to body dissatisfaction (*r* = 0.284), such that people who had more adaptive emotional regulation presented lower scores in body dissatisfaction. Additionally, greater problematic use of social media, in all its dimensions, was related to greater body dissatisfaction (*r* = 0.269) (see Table 2). Finally, age was negatively related to cognitive emotion regulation strategies (*r* between −0.054 and −0.208) and to lower problematic social media use (*r* = −0.310). Moreover, older age was related to lower emotional distress (*r* = −0.286), lower stress (*r*= −0.288), and lower body dissatisfaction (*r* = −0.231).

Finally, from a simple mediation analysis using ordinary least squares path analysis, the mediating roles of problematic use of social media and emotional regulation strategies were explored in the relationship between emotional distress and body dissatisfaction.

**Figure 1:**
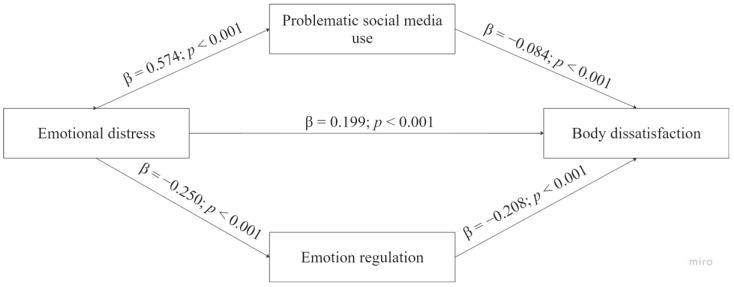
Diagram of the mediation analysis model.

As can be seen in Figure 1, participants with greater emotional distress showed higher problematic social media use (a = 0.574) and lower emotion regulation (a = −0.250). Likewise, higher problematic social media use and emotional regulation were negatively related to body dissatisfaction (b = −0.084 and b = −0.208, respectively). As mentioned above, a bootstrap confidence interval for the indirect effect based on 10,000 bootstrap samples showed the indirect effect of problematic social media use (ab = 0.048, 95% CI [0.033, 0.064]) and emotion regulation (ab = 0.052, 95% CI [0.039, 0.066]). The total effect of emotional distress on body dissatisfaction of c’ = 0.199 was statistically significant (*p* < 0.001, 95% CI [0.267, 0.331]) (see Table 3).

## 4. Discussion

The present study had three main objectives. Firstly, to find sex differences in emotional distress (i.e., depression, anxiety, and stress), emotional regulation, social media use, and body dissatisfaction. Secondly, to study the relationship between the four variables: emotional regulation, problematic use of social media, body dissatisfaction, and emotional distress. Finally, to examine the mediating role of emotional regulation and social media use in the relationship between emotional distress and body dissatisfaction.

On one hand, the results showed that women score significantly higher than men on body dissatisfaction, anxiety, depression, stress, emotion regulation, and problematic social media use. Women’s higher body dissatisfaction scores could be closely related to the constant change in female beauty standards [40], which drives women to adapt to every fashion. As a result, body satisfaction becomes a difficult and unlikely goal [65]. It is also suggested that body dissatisfaction may be one of the etiological roots of EDs. Thus, the low likelihood of achieving an ideal body would increase the risk of EDs [18].

Concerning anxiety, depression, and stress, more women than men are diagnosed with anxiety and depression. According to the Spanish Ministry of Health [66], the prevalence of depression in women was 5.9% compared to 2.3% in men and, for anxiety, the prevalence was 8.8% in women compared to 4.5% in men. This preponderance of women in these conditions is explained by an interplay of a range of factors, including biological, social (e.g., structural gender inequalities), and cultural (e.g., gender roles) [67,68]. In terms of emotional regulation, as other studies have found [47,69], women scored significantly higher than men on the global scale, as well as on the subscales of Rumination, Catastrophizing, Positive Refocusing, and Acceptance. Studies have reported that women are more likely to use a wider range of emotion regulation strategies than men [70]. These findings might be explained by gender roles, given that men tend to engage more in problem-solving techniques, whereas women tend to use more passive and internalized responses to their emotions, such as rumination and catastrophizing [71,72]. Similarly, men are more likely to engage in emotional avoidance and impulsive behavior [71].

Moreover, women presented more problematic use of social media than men. This difference could be due to several factors, including gender roles and sex differences in socialization [73,74]. The need for group membership, acceptance, and social validation through physical appearance or the ideal body could also lead women to turn to social media in search of positive reinforcers (e.g., likes, comments, or social media interactions) [9,65]. In fact, most studies on body image and social media have focused on the young and/or adolescent female population [10]. This is largely due to the vulnerability of this population to social pressures to achieve a stereotype of beauty [7,17,26]. Consistent with these findings, Papageorgiou et al. found similar results in their qualitative study [75]. Their study used a sample of adolescent females who identified body image as one of their primary concerns. They also found that appearance comparisons exacerbated adolescent girls’ body image concerns and influenced their efforts to change their appearance and seek validation through social media [75].

On the other hand, correlational analysis between variables has shown a strong relationship between emotional distress (i.e., depression, anxiety, and stress) and emotion regulation strategies, underlining the association between anxiety and rumination and depression and catastrophizing. These findings are consistent with previous studies showing that rumination [76] and catastrophizing [77] are transdiagnostic variables observed in multiple psychopathologies, such as post-traumatic stress disorder, obsessive-compulsive disorder, anxiety disorders, depressive disorders, and body dysmorphic disorder [78,79].

Regarding age, it was found that age was negatively related to emotional distress (i.e., anxiety, depression, and stress) and body dissatisfaction. This finding is consistent with some studies suggesting that life experience and maturation help people cope with stressful situations and social pressures [80,81]. Conversely, some authors deny that age is a determining factor in suffering from emotional distress or body dissatisfaction because emotional discomfort and body dissatisfaction are complex realities that involve more than one factor [10,18,82]. Other authors insist that emotional distress increases with age due to the increase in responsibilities and potential major age-associated life events, such as diagnosis of illness, death of loved ones, etc. [83]. Likewise, age is negatively correlated with problematic social media use. In fact, in Spain, more young people use social media than older adults. Specifically, 63.4% of people aged 45–54 and 47.1% aged 55–64 use social media [55]. However, as mentioned above, the studies were mainly conducted with young people [9,10,53]. Therefore, there is no information to contrast these results with, so it would be interesting to study the profiles and needs of adult users.

Finally, the mediation analysis showed that the relationship between emotional distress and body dissatisfaction was mediated by problematic social media use and emotion regulation strategies. Several previous studies have shown that anxiety [16], depression [13,67], and stress [1] are associated with greater body dissatisfaction.

Furthermore, the association between social media use and body dissatisfaction has been widely reported in previous studies [1,40]. Studies have found that the pressure to look perfect on social media leads to body dissatisfaction [7], as social media is a space where social comparison is common [14] and where flattering images and videos [42,43], such as fitspiration, travel, and holidays, are posted.

It is well-known and proven that social media allows individuals to reflect a desired image of themselves. In the process of creating a public image, users may spend a great deal of time taking photos and editing them with filters and applications [8,65]. This process is known as self-presentation [84]. Self-presentation mechanisms promote self-objectification or the perception of oneself as an object rather than a person. In a study of Instagram use and the internalization of beauty standards, Feltman and Szymanski found that the time spent on the platform predicted body satisfaction, body self-checking, and appearance comparisons [85]. In this regard, Ahadzadeh et al. suggest a new emerging issue due to the power of social media to exacerbate self-objectification, as it leads people to view themselves through an observer’s perspective. The disconnection between online identity and real life may increase appearance anxiety [86,87].

In fact, given the important role of physical appearance in social media [48], one of the most studied variables explaining the relationship between social media use and body dissatisfaction is the seemingly authentic images representing body ideals [14]. Kleemans et al. concluded in their study that manipulated Instagram images had a negative effect on adolescent girls’ body image, and this effect was moderated by social comparison [65]. In other words, the posting of content that depicts a seemingly perfect lifestyle and canonical beauty standards perpetuates a cycle of social comparison that simultaneously has a negative impact on body satisfaction and quality of life [9,11]. Similarly, accounts with optimum engagement rates (e.g., Kylie Jenner, Cristiano Ronaldo, Zendaya, Kim Taehyung, etc.) have been shown to significantly influence the behavior and consumption of their followers [10,52].

In this context, emotion regulation is one of the most studied transdiagnostic variables in mental health problems [44,45], including substance addictions [84] and behavioral addictions [88]. Along these lines, training in emotion regulation strategies could help reduce the impact of emotional distress on body dissatisfaction [89]. Social media users also use these platforms as a coping strategy; that is, a means of dealing with distressful situations [51,90,91] and, therefore, they may play a role in issues related to body image and social comparison [9,14,24]. Thus, adaptive emotion regulation strategies could help social media users to effectively manage their cognitions and ideas about social media content [48]. In other words, emotion regulation strategies would allow users to appropriately manage and reduce the pressures of social comparison and beauty standards [40,81]. In 2020, LeBlanc indicated that emotion training in the intervention group was associated with decreases in depressive symptomatology, worry, and suppression, and concurrent increases in overall mental well-being [89]. This program has been shown to be effective with people who have emotional adjustment problems. Therefore, people with negative body image may benefit from this type of emotionally focused intervention [44,52,89].

Many studies are consistent with the findings of this article. On the one hand, at the level of the psychological impact of social media, many authors point out that psychological distress is associated with the use of social media [16,53,91]. On the other hand, some studies have shown that social media is used as a coping strategy in stressful situations [48,50]. Furthermore, some studies highlight the importance of emotion regulation as a possible protective factor in social media use [7,48,50]. Finally, scientific evidence points to the influence of social media on body image and body evaluation [1,39,52,65]. However, there is still a long way to go in this area of research, as longitudinal and experimental studies and intervention designs are scarce [1,39]. This is due to the foundational nature of current research on social media and body image. A clear example of this is the lack of consensus on the definition of problematic social media use, the poor definition of the type of use and content offered by each social platform, or the ever-changing aesthetic trends and target audiences. Social media and the concept of beauty are ever-changing constructs because they depend mainly on sociocultural, economic, and political factors that are difficult to predict. Logically, the primary goal of researchers is to establish a theoretical foundation. Fortunately, in the face of these knowledge gaps, social and scientific awareness of the issue is growing [18,44,90].

Nevertheless, these results should be interpreted considering several limitations. Firstly, this is a cross-sectional study, so a longitudinal follow-up is required to examine causal relationships between these variables. Secondly, the role of age in the relationship between the target variables was not investigated. Finally, the instrument to assess body dissatisfaction was the nine-item subscale of the EDI-II [61]. Although this subscale has been shown to be reliable in this study, it does not examine the phenomenon of body dissatisfaction as a whole. Instead, it serves as a very useful screening test that should be contrasted with other assessment tools and techniques in order to evaluate body dissatisfaction. In addition, all the instruments used were self-reports, which makes the data susceptible to many biases, including social desirability. Future studies should use complementary tests or qualitative methods to observe the relationship between the studied variables.

## 5. Conclusions

In short, the study found that women scored higher on emotional distress, body dissatisfaction, emotional regulation, and problematic use of social media. Additionally, a relationship was observed between emotional regulation, emotional distress, body dissatisfaction, and problematic use of social media. Finally, the relationship between emotional distress and body dissatisfaction was mediated by the problematic use of social media and emotional regulation. These findings highlight the importance of emotional regulation and social media use to effectively address and prevent body dissatisfaction and, thus, EDs. Future works should include prospective studies and qualitative analyses of the impact of mental health on body satisfaction and the role of social media and emotion regulation strategies.

## Figures and Tables

**Table 1:** Descriptive statistics based on sex.

	Total (*N* = 2520)	Men (*n* = 1231)	Women (*n* = 1289)	*p*-Value	Cohen’s *d*
Emotional distress	11.10 (11.94)	8.48 (9.98)	13.61 (13.07)	<0.001	0.442
Depression	3.58 (4.59)	2.77 (3.92)	4.35 (5.02)	<0.001	0.351
Anxiety	2.70 (3.75)	1.94 (3.07)	3.42 (4.17)	<0.001	0.405
Stress	4.82 (4.57)	3.77 (3.92)	5.83 (4.91)	<0.001	0.464
Cognitive emotion regulation strategies	60.52 (8.36)	60.88 (7.75)	60.18 (8.89)	0.037	0.084
Putting into perspective	6.10 (2.18)	6.02 (2.17)	6.17 (2.18)	0.074	0.069
Positive refocusing	5.02 (2.09)	4.92 (2.08)	5.12 (2.10)	0.018	0.096
Positive reappraisal	6.45 (2.22)	6.39 (2.18)	6.49 (2.25)	0.301	0.045
Acceptance	6.12 (2.18)	6.04 (2.18)	6.21 (2.17)	0.049	0.078
Refocusing on planning	6.04 (2.12)	6.06 (2.12)	6.03 (2.11)	0.780	0.014
Self-blame	4.22 (1.91)	4.15 (1.88)	4.29 (1.94)	0.065	0.073
Blaming others	3.77 (1.78)	3.75 (1.72)	3.79 (1.85)	0.568	0.022
Rumination	5.21 (2.07)	4.94 (1.95)	5.47 (2.15)	<0.001	0.258
Catastrophizing	4.01 (1.99)	3.71 (1.86)	4.29 (2.08)	<0.001	0.294
Body dissatisfaction	17.22 (8.90)	16.62 (6.47)	17.81 (5.24)	<0.001	0.202
Problematic social media use	45.80 (16.47)	43.19 (15.43)	48.29 (17.04)	<0.001	
Obsession	16.90 (6.57)	6.32 (0.18)	6.74 (0.19)	<0.001	
Lack of control	11.20 (4.72)	4.38 (0.12)	4.91 (0.14)	<0.001	
Excessive use	17.69 (6.29)	5.73 (0.16)	6.59 (0.18)	<0.001	

**Table 2:** Bivariate correlations.

	1	2	3	4	5	6	7	8	9	10	11	12	13	14	15	16	17	18	19
Emotional distress	-																		
2. Depression	0.931 ***	-																	
3. Anxiety	0.913 ***	0.783 ***	-																
4. Stress	0.930 ***	0.786 ***	0.780 ***	-															
5. Cognitive Emotion regulation Strategies	−0.357 ***	−0.375 ***	−0.315 ***	−0.298 ***	-														
6. Putting into perspective	−0.031	−0.046 *	−0.036	−0.006	0.625 ***	-													
7. Positive refocusing	−0.029	−0.051 **	0.007	−0.031	0.516 ***	0.372 ***	-												
8. Positive reappraisal	−0.086 ***	−0.122 ***	−0.070 ***	−0.044 *	0.706 ***	0.572 ***	0.396 **	-											
9. Acceptance	0.140 ***	0.136 ***	0.116 ***	0.134 ***	0.341 ***	0.359 ***	0.225 ****	0.395 ***	-										
10. Refocusing on planning	0.026	−0.013	0.025	0.059 **	0.589 ***	0.462 ***	0.418 ***	0.653 ***	0.353 ***	-									
11. Self-blame	0.391 ***	0.382	0.347 ***	0.354 ***	−0.286 ***	0.092 ***	0.051*	0.074 ***	0.383 ***	0.173 ***	-								
12. Blaming others	0.260 ***	0.233	0.253 ***	0.237 ***	−0.298 ***	0.097 ***	0.130 ***	0.032	0.062 **	0.081 ***	0.151 ***	-							
13. Rumination	0.418 ***	0.408	0.351 ***	0.394 ***	−0.196 ***	0.173 ***	0.168 ***	0.205 ***	0.468 ***	0.299 ***	0.509 ***	0.234 ***	-						
14. Catastrophizing.	0.474 ***	0.470 ***	0.438 ***	0.407 ***	−0.439 ***	0.025	0.083 ***	0.008	0.195 ***	0.100 ***	0.416 ***	0.400 ***	0.508 ***	-					
15. Body dissatisfaction	0.347 ***	0.328 ***	0.312 ***	0.321 ***	−0.284 ***	−0.082 ***	−0.071 ***	−0.150 ***	0.042 *	−0.072 ***	0.216 ***	0.140 ***	0.223 ***	0.263 ***	-				
16. Problematic social media use	0.419 ***	0.384 ***	0.403 ***	0.378 ***	−0.235 ***	0.005	0.029	−0.040 *	0.094 ***	−0.001	0.255 ***	0.240 ***	0.279 ***	0.330 ***	0.269 ***	-			
17. Obsession	0.379 ***	0.348 ***	0.378 ***	0.330 ***	−0.287 ***	−0.054 **	0.013	−0.113 ***	0.006	−0.067 ***	0.226 ***	0.265 ***	0.198 ***	0.310 ***	−0.241 ***	0.918 ***	-		
18. Lack of control	0.408 ***	0.380 ***	0.392 ***	0.364 ***	−0.237 ***	−0.004	0.029	−0.035	0.089 **	0.005	0.255 ***	0.223 ***	0.292 ***	0.334 ***	0.265 ***	0.943 ***	0.815 ***	-	
19. Excessive use	0.387 ***	0.352 ***	0.357 ***	0.366 ***	−0.162 ***	0.053 **	0.032	0.015	0.151 ***	0.043 *	0.236 ***	0.188 ***	0.298 ***	−0.293 ***	0.261 ***	0.944 ***	0.782 ***	0.857 ***	
20. Age	−0.286 **	−0.237 **	−0.271 **	−0.288 **	0.163 **	0.069 **	0.035	−0.006	−0.054 **	−0.056 **	−0.170 **	−0.135 **	−0.198 **	−0.208 **	−0.231 **	0.310 **	0.248 **	0.338 **	0.305 **

* *p* ≤ 0.05. ** *p* ≤ 0.01. *** *p* ≤ 0.001.

**Table 3:** Effects of the mediation analyses: the mediating role of emotional regulation and problematic social media use in the relationship between emotional distress and body dissatisfaction.

	*R* ^2^	β	*p*-Value	*SE*	LL	UL
Direct effect						
Emotional distress—Body dissatisfaction		0.199	<0.001	0.018	0.164	0.234
ARS—Body dissatisfaction		0.084	<0.001	0.012	0.059	0.108
CERQ—Body dissatisfaction		−0.208	<0.001	0.024	−0.255	−0.161
Emotional distress—ARS	0.173	0.574	<0.001	0.025	0.525	0.622
Emotional distress—CERQ	0.127	−0.250	<0.001	0.0130	−0.275	−0.224
Indirect effects						
Emotional distress—ARS—Body dissatisfaction		0.048	-	0.008	0.033	0.064
Emotional distress—CERQ—Body dissatisfaction		0.052	-	0.007	0.039	0.066
Total effects	0.120	0.299	<0.001	0.016	0.267	0.331

Note. SE = Standard error; LL = lower limit; UL = upper limit; CERQ = cognitive emotion-regulation questionnaire; ARS = Problematic Social Media Use [Spanish acronym: Adicción a Redes Sociales].

## Data Availability

The data used in the study can be made available on requests addressed to the corresponding author.
